# A latent profile analysis of hemodialysis nurses’ knowledge, attitude, and practice toward medical device alarm fatigue in Henan, China

**DOI:** 10.3389/fpubh.2026.1852268

**Published:** 2026-06-09

**Authors:** Liyang Zhu, Meisu Lu, Zhen Chen, Xiaoxing Wang, Xiaoyang Wang

**Affiliations:** 1 Department of Cardiovascular Surgery, The First Affiliated Hospital of Zhengzhou University, Zhengzhou, China; 2 Health Education Center, The Third Affiliated Hospital of Zhengzhou University, Zhengzhou, China; 3 Blood Purification Center, The First Affiliated Hospital of Zhengzhou University, Zhengzhou, China

**Keywords:** alarm fatigue, hemodialysis, knowledge-attitude-practice, latent profile analysis, nurses

## Abstract

**Background:**

This study aims to investigate the characteristic variances in the knowledge, attitude, and practice (KAP) levels concerning medical device alarm fatigue among hemodialysis nurses and analyze the influencing factors.

**Methods:**

A cross-sectional study was conducted using convenience sampling. Between May and June 2023, a total of 1,187 hemodialysis nurses from 126 units in Henan Province completed the survey. After data cleaning, 1,154 nurses were included in the final analysis. Research instruments included a general information questionnaire, a KAP questionnaire on medical device alarm fatigue for hemodialysis nurses, and a resilience scale. Latent profile analysis was carried out to categorize the KAP levels. Single-factor analysis and multinomial logistic regression were employed to identify influencing factors.

**Results:**

The KAP levels were divided into three categories: the medium-level group (73.9%), the knowledge deficit group (10.3%), and the high-level group (15.8%). Compared with the high-level group, nurses aged under 30 years and those with 1–5 years of experience had higher odds of being in the medium-level group. Nurses who had received training on medical device alarm-related knowledge and those with higher resilience scores had lower odds of being in the medium-level group or the knowledge-deficit group. Furthermore, compared with the high-level group, nurses working in higher-grade hospitals, those who had participated in adverse event analysis, and those with higher resilience scores had lower odds of being in the knowledge-deficit group.

**Conclusion:**

The KAP levels regarding medical device alarm fatigue among hemodialysis nurses demonstrate heterogeneity. Nursing managers may consider designing targeted interventions based on the influencing factors of different categories to improve the KAP levels. Future intervention studies incorporating objective measures are needed to confirm these findings.

## 1 Introduction

Medical device alarm fatigue refers to a phenomenon in which healthcare staff become desensitized or unresponsive to alarms after prolonged exposure (1). Studies have shown that the level of medical device alarm fatigue among hemodialysis nurses is relatively high (2). Persistent alarm fatigue not only exacerbates job burnout among nurses but also impairs their alarm sensitivity (3, 4), leading to ignored warnings, compromised monitoring, and significant threats to patient safety (5). Recognizing this risk, the Chinese Hospital Association’s “Patient Safety Goals (2019 Edition)” explicitly listed “strengthening the safety and alarm management of medical equipment” as a top priority (6).

While a substantial body of alarm fatigue research exists, it predominantly focuses on Intensive Care Unit (ICU) settings (7, 8). Hemodialysis equipment alarms possess distinct particularities compared to ICU monitors (9). Beyond monitoring and warning functions, hemodialysis machines perform the core therapeutic task of blood purification, meaning that alarm responses directly impact treatment continuity. Furthermore, hemodialysis nurses often manage multiple machines independently in a closed environment, with fewer backup staff than typical ICU settings. Consequently, the knowledge, attitudes, and practices (KAP) of hemodialysis nurses—as the primary users and alarm handlers—are critical determinants of patient safety. However, existing studies on alarm fatigue among hemodialysis nurses (10) have predominantly employed variable-centered approaches (e.g., multiple regression) that examine relationships between aggregate scores. While valuable for identifying average effects, these methods assume homogeneity across individuals and cannot identify distinct subgroups of nurses who may share specific KAP patterns. For example, a variable-centered analysis might conclude that “knowledge is generally moderate,” obscuring the possibility that some nurses have high practical skills but poor theoretical knowledge, while others have adequate knowledge but unfavorable attitudes. This distinction matters because different subgroups may require fundamentally different interventions. To our knowledge, no previous study has employed a person-centered analytical technique to classify hemodialysis nurses into subgroups based on their patterns of knowledge, attitude, and practice regarding device alarms. This gap is significant because tailored interventions—matching the specific needs of each subgroup—are likely more efficient and effective than one-size-fits-all approaches.

Latent profile analysis (LPA) is an advanced, person-centered statistical method that classifies individuals into unobserved subgroups (latent profiles) based on their responses to a set of continuous variables (11). This approach allows for the identification of homogeneous subpopulations within a larger heterogeneous group, maximizing between-profile differences while ensuring within-profile similarity (12). Applying LPA to KAP data can reveal nuanced patterns that variable-centered analyses might obscure, such as a group with high practical skills but poor theoretical knowledge, or vice versa.

The primary innovation of this study lies in its application of LPA to elucidate the heterogeneity in KAP levels concerning medical device alarm fatigue among hemodialysis nurses. Furthermore, resilience was selected *a priori* as a key moderator based on its established role in healthcare stress adaptation (13). By moving beyond describing average scores to identifying distinct nurse profiles, this study addresses a significant gap in the literature. The findings will provide a nuanced evidence base for nursing managers to develop precisely targeted, profile-specific interventions—such as tailored training for the “knowledge-deficit” group or resilience-building programs for those with high knowledge but unfavorable attitudes—rather than applying a one-size-fits-all approach. This person-centered strategy is crucial for effectively mitigating alarm fatigue and enhancing patient safety in hemodialysis units.

## 2 Materials and methods

### 2.1 Research subjects and sampling procedure

This cross-sectional study employed a multistage convenience sampling strategy. The sampling frame consisted of all 178 hemodialysis units registered with the Henan Provincial Nursing Quality Control Center as of April 2023.

Stage 1 (Unit selection): From the 178 eligible units, we aimed to recruit approximately 120–130 units based on feasibility considerations (estimated 10–15 nurses per unit). Using convenience sampling, we contacted the nursing directors of 140 units via the provincial nursing quality control network. A total of 126 units agreed to participate (response rate: 90.0%). These included 89 tertiary hospitals, 34 secondary hospitals, and 3 primary hospitals, geographically distributed across 17 prefecture-level cities in Henan Province.

**Figure 1:**
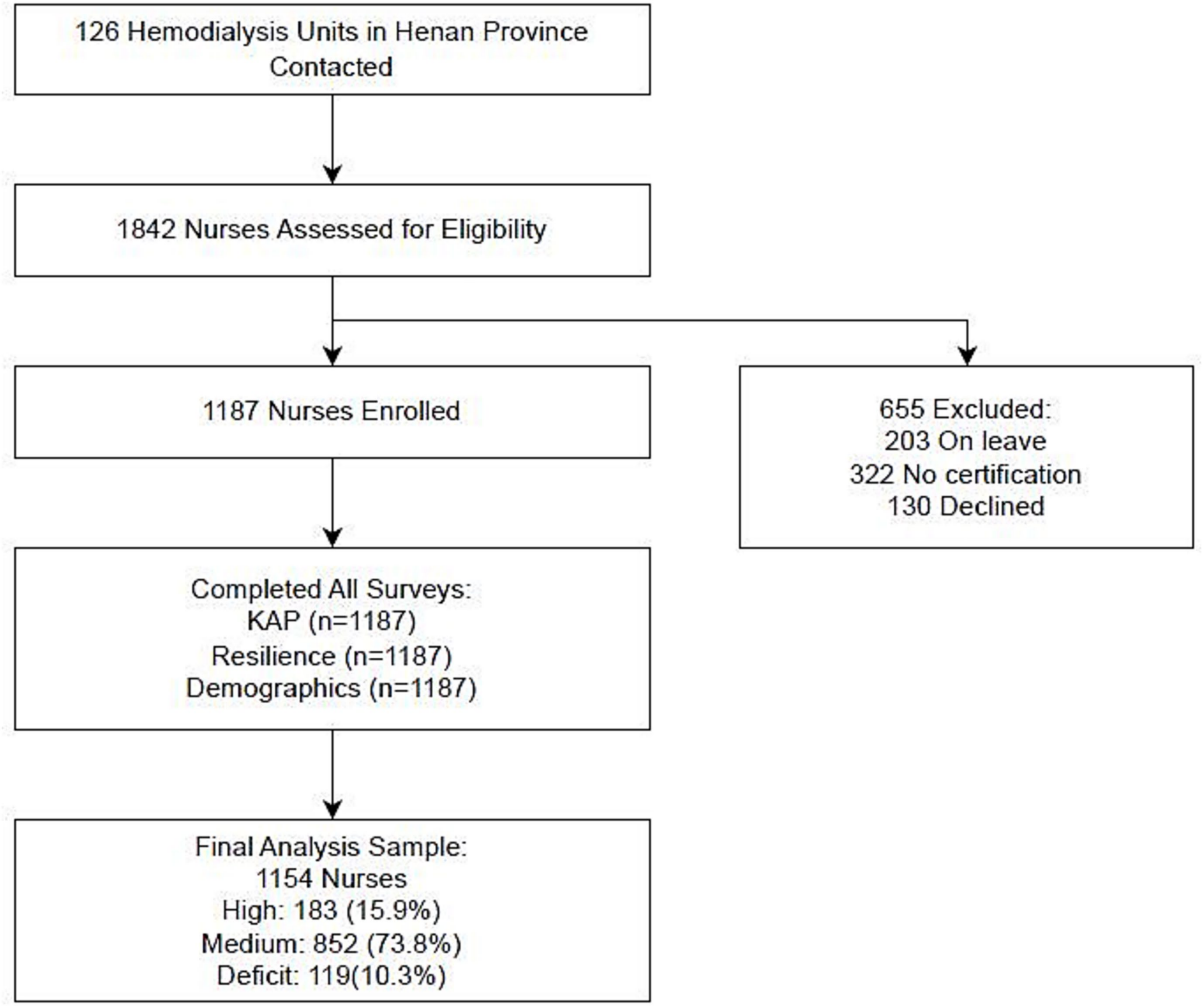
Participant selection process for the cross-sectional study of alarm fatigue among hemodialysis nurses in Henan Province, China (May–June 2023). From 126 initially contacted hemodialysis units, 1,187 certified nurses met inclusion criteria and completed all survey instruments. Thirty-three responses were excluded during data cleaning, yielding a final analytic sample of 1,154 nurses categorized into three KAP profiles through latent profile analysis.

Stage 2 (Participant recruitment): Within each participating unit, all nurses who met the inclusion criteria were invited to participate. The inclusion criteria were as follows: ① holding a valid nursing license; ② certification in hemodialysis care (e.g., completion of a nationally accredited training program); ③ ≥ 3 months of hemodialysis unit experience. Certified nurses were prioritized to ensure standardized exposure to device alarms (14). The exclusion criteria were as follows: nurses on further training, internship, standardized training, and those who were on leave during the survey period. This flowchart provides a clear and structured overview of the research process (Figure 1).

### 2.2 Research tools

#### 2.2.1 General information

The general information questionnaire was designed by the researchers based on a literature review. It included age, gender, marital status, number of children, educational level, professional title, hospital grade and nature, work experience, monthly income, whether working the continuous renal replacement therapy (CRRT) shift, whether being a hemodialysis specialist nurse, whether participating in medical device alarm-related knowledge training, and whether participating in the analysis of adverse events related to medical device alarms.

#### 2.2.2 Knowledge, attitude, and practice questionnaire on medical device alarm fatigue for Hemodialysis nurses

This questionnaire was developed in China and is used to investigate the current status of knowledge, attitude, and practice regarding medical device alarm fatigue among hemodialysis nurses. The questionnaire consists of three dimensions: knowledge, attitude, and practice. The knowledge dimension has nine items, the attitude dimension has nine items, and the practice dimension has 15 items. All dimensions are scored using a 5-point Likert scale. The higher the scores of the three dimensions, the higher the level of knowledge, attitude, and practice regarding alarms and alarm fatigue among hemodialysis nurses. The total score range of the KAP questionnaire is 33–165 points. The Cronbach’s *α* coefficient of the questionnaire is 0.822, and the content validity is 0.902, indicating good reliability and validity (15).

All data collected in this study were self-reported by participants. The KAP questionnaire and the resilience scale rely on participants’ own perceptions, recollections, and subjective judgments. No objective measures (e.g., direct observation of alarm response behaviors, physiological measures of fatigue, or objective knowledge tests) were employed. This self-report methodology carries inherent biases that are addressed in the Limitations section.

#### 2.2.3 Resilience scale

This scale was developed by American psychologists Connor and Davidson and was revised and translated into Chinese and colleagues in China (16). The scale has a Cronbach’s *α* coefficient of 0.91. It consists of 25 items divided into three dimensions: tenacity (13 items), self-reliance (8 items), and optimism (4 items). The items are scored using a 5-point Likert scale (0–4 points per item), with a total score ranging from 0 to 100 points. A higher score indicates a higher level of resilience.

### 2.3 Data collection method

Approval and pre-survey preparation: After obtaining ethical approval (No. 2025-KY-0591-003) and administrative approval from the nursing departments of participating hospitals, we initiated data collection. A designated coordinator from each hospital’s hemodialysis unit was trained via a standardized 30-min online session covering the study purpose, questionnaire administration, and data quality protocols.

Online survey platform: Data were collected using the Questionnaire Star platform (Changsha Ranxing Information Technology Co., Ltd., China), a HIPAA-compliant tool for health research. The platform provided enterprise-level security features, including data encryption and access controls.

Measures to prevent duplicate participation: To ensure each nurse completed the survey only once, we implemented the following technical safeguards: (1) IP address locking—the survey link could only be accessed from IP addresses registered to participating hospital’s internal networks; (2) Device fingerprinting—the platform recorded unique device identifiers and blocked re-submission from the same device; (3) Unique one-time login links—each eligible nurse received a personalized survey link that expired immediately after submission; (4) Completion tracking—the platform logged completion status and prevented re-submission after final completion; (5) Manual cross-checking—we cross-referenced participant IDs, timestamps, and device fingerprints to identify any potential duplicates. No duplicate submissions were detected.

Survey administration: The questionnaire included a unified set of instructions explaining the purpose, voluntary nature, and anonymity of the study. Participants were required to acknowledge electronic informed consent before accessing the questionnaire. The platform was configured with answer restrictions and a minimum completion time of 8 min.

Data cleaning: Invalid questionnaires were defined as those with: (1) completion time < 5 min, (2) identical responses across all items (straight-lining), or (3) logically contradictory answers. A total of 33 questionnaires were excluded based on these criteria.

### 2.4 Statistical methods

Data analysis was conducted using SPSS 27.0 and Mplus 8.0 software. The reliability of the research tools in this study was confirmed by calculating Cronbach’s alpha. The KAP questionnaire showed good internal consistency with a Cronbach’s *α* of 0.822, and the Resilience Scale (CD-RISC) also demonstrated high reliability with a Cronbach’s α of 0.91.

Model selection for LPA was guided by a combination of statistical fit indices and theoretical interpretability. While the 4-profile solution showed improved fit indices (lower AIC, BIC, aBIC) compared to the 3-profile model, one of its categories comprised only 2.7% of the sample (*n* = 32), falling below the recommended threshold of 5% for meaningful subgroup identification. Such a small group may represent statistical noise rather than a clinically or practically distinct profile, risking overfitting and reduced generalizability. Additionally, the 3-profile solution achieved high entropy (0.995), indicating excellent classification accuracy, and aligned with prior research on KAP heterogeneity in healthcare populations (14). Consistent with COR theory, resilience was specified as a covariate during initial model formulation. Thus, we prioritized parsimony and interpretability by retaining the 3-profile model.

The normality of continuous variables was assessed using the Kolmogorov–Smirnov test. Count data were expressed as frequencies and percentages, and measurement data were expressed as means and standard deviations. Comparisons between the latent profile groups on demographic and work-related characteristics were made using the χ^2^ test or Kruskal-Wallis H test, as appropriate. To explore the influencing factors associated with the KAP latent profiles, multinomial logistic regression analysis was used. Prior to regression analysis, Variance Inflation Factors (VIF) were examined to confirm the absence of multicollinearity among independent variables (all VIF < 10). For the regression analysis, the independent variables were coded as follows: Age: <30 (Reference: >50); Gender: male = 0, female = 1 (Reference: female); Education level: associate degree or below, bachelor’s degree (Reference: master’s degree or above); Professional title: nurse, senior nurse, head nurse (Reference: deputy chief nurse or above); Hospital grade: tertiary, secondary (Reference: primary); Work experience in hemodialysis: 1–5 years, 6–10 years, 11–15 years (Reference: ≥16 years); Working the CRRT shift: yes = 1, no = 0 (Reference: no); Being a hemodialysis specialist nurse: yes = 1, no = 0 (Reference: no); Participating in alarm-related knowledge training: yes = 1, no = 0 (Reference: no); Participating in adverse event analysis of alarms: yes = 1, no = 0 (Reference: no); Psychological resilience: raw scores were used as a continuous variable. A *p*-value less than 0.05 was considered statistically significant, with a significance level α = 0.05.

## 3 Results

### 3.1 General information of the study subjects

A total of 1,187 questionnaires were collected. After excluding 33 invalid responses, 1,154 valid questionnaires were retained (effective response rate: 97.2%). The sample included 188 male and 966 female nurses (Note: gender distribution adjusted to *n* = 1,154). The mean total score of psychological resilience was 80.9 ± 10.5 (see Table 1 for details).

**Table 1:** Univariate analysis of general information and potential profiles of medical device alarm fatigue KAP.

Item	Medium-level group (*n* = 852, 73.9%)	Knowledge-deficit group (*n* = 119, 10.3%)	High-level group (*n* = 183, 15.8%)	Test statistic	*P*-value
Age [*n*(%)]				185.751^1^	<0.001
<30	342(70.8%)	110(22.8%)	31(6.4%)		
31–40	439(81.9%)	9(1.7%)	88(16.4%)		
41–50	77(55.8%)	2(1.4%)	59(42.8%)		
>50	18(60.0%)	1(3.3%)	11(36.7%)		
Gender [*n*(%)]				17.677^2^	<0.001
Male	130(69.1%)	35(18.7%)	23(12.2%)		
Female	722(74.7%)	84(8.7%)	160(16.6%)		
Hospital grade				76.76	<0.001
Tertiary	670(76.3%)	52(5.9%)	156(17.8%)		
Secondary	173 (68.4%)	57 (22.5%)	23 (9.1%)		
Primary	9 (42.9%)	10 (47.6%)	2 (9.5%)		
Marital status [*n*(%)]				2.458	0.601
Married	612(74.8%)	79(9.7%)	12(15.5%)		
Unmarried	261(71.5%)	43(11.8%)	61(16.7%)		
Divorced	3(75.0%)	0(0%)	1(25.0%)		
Number of children [*n*(%)]				5.757	0.056
0	244(76.2%)	36(11.3%)	40(12.5%)		
1	321(68.6%)	60(12.8%)	87(18.6%)		
2	297(78.4%)	25(6.6%)	57(15.0%)		
>2	14(70.0%)	1(5.0%)	5(25.0%)		
Educational level [*n*(%)]				76.708	<0.001
Associate degree or below	235(64.6%)	81(22.3%)	48(13.1%)		
Bachelor’s degree	636(78.3%)	39(4.8%)	137(16.9%)		
Master’s degree or above	5(45.5%)	2(18.1%)	4(36.4%)		
Professional title [*n*(%)]				209.818	<0.001
Nurse	151(55.3%)	103(37.7%)	19(7.0%)		
Senior nurse	339(82.3%)	15(3.6%)	58(14.1%)		
Head nurse	361(77.3%)	3(0.6%)	103(22.1%)		
Deputy chief nurse or above	25(71.4%)	1(1.9%)	9(25.7%)		
Hospital level [*n*(%)]				76.761^1^	<0.001
Tertiary	670(76.3%)	52(5.9%)	156(17.8%)		
Secondary	197(68.4%)	60(20.8%)	31(10.8%)		
Primary	9(42.9%)	10(47.6%)	2(9.5%)		
Hospital nature [*n*(%)]				1.893^2^	0.394
Public	725(74.5%)	95(9.8%)	153(15.7%)		
Private	127(70.6%)	24(12.6%)	29(16.8%)		
Years of experience in hemodialysis work [*n*(%)]				66.385^1^	<0.001
1–5	397(75.5%)	80(15.2%)	49(9.3%)		
6–10	272(72.5%)	37(9.9%)	66(17.6%)		
11–15	148(76.3%)	3(1.5%)	43(22.2%)		
≥16	35(61.4%)	2(3.5%)	20(35.1%)		
CRRT shift? [*n*(%)]				18.804	<0.001
Yes	576(72.2%)	103(12.9%)	119(14.9%)		
No	276(77.1%)	16(4.5%)	64(17.9%)		
Hemodialysis specialist nurse? [*n*(%)]				7.272	0.025
Yes	755(72.5%)	111(10.7%)	175(16.8%)		
No	97(82.9%)	8(6.8%)	12(10.3%)		
Monthly income [*n*(%)]				5.396	0.067
<3,000 RMB	142(89.9%)	1(0.6%)	15(9.5%)		
3,000–6,000 RMB	542(69.0%)	116(14.8%)	128(16.2%)		
6,000–10,000 RMB	124(77.0%)	3(1.9%)	34(21.1%)		
>10,000 RMB	44(846%)	2(3.8%)	6(11.5%)		
Participated in alarm-related knowledge training? [*n*(%)]				17.722	<0.001
Yes	656(71.0%)	102(11.0%)	166(18.0%)		
No	196(83.8%)	17(7.3%)	21(8.9%)		
Participated in adverse event analysis? [*n*(%)]				19.205	<0.001
Yes	323(80.1%)	21(5.2%)	59(14.7%)		
No	529(70.5%)	98(12.9%)	123(16.6%)		
Total score of psychological resilience (points)	78.9 ± 10.5	78.4. ± 5.9	91.6 ± 5.2	299.906	<0.001

^1^H value; ^2^χ^2^ value; ^3^Fisher’s exact probability method.

### 3.2 Knowledge-attitude-practice level of medical device alarm fatigue among hemodialysis nurses

The total KAP score for medical device alarm fatigue among hemodialysis nurses was 116.9 ± 14.4. Specifically, the knowledge dimension scored 33.1 ± 8.4, the attitude dimension scored 28.0 ± 2.9, and the practice dimension scored 55.8 ± 7.7.

### 3.3 Latent profile analysis of KAP level of medical device alarm fatigue

**Figure 2:**
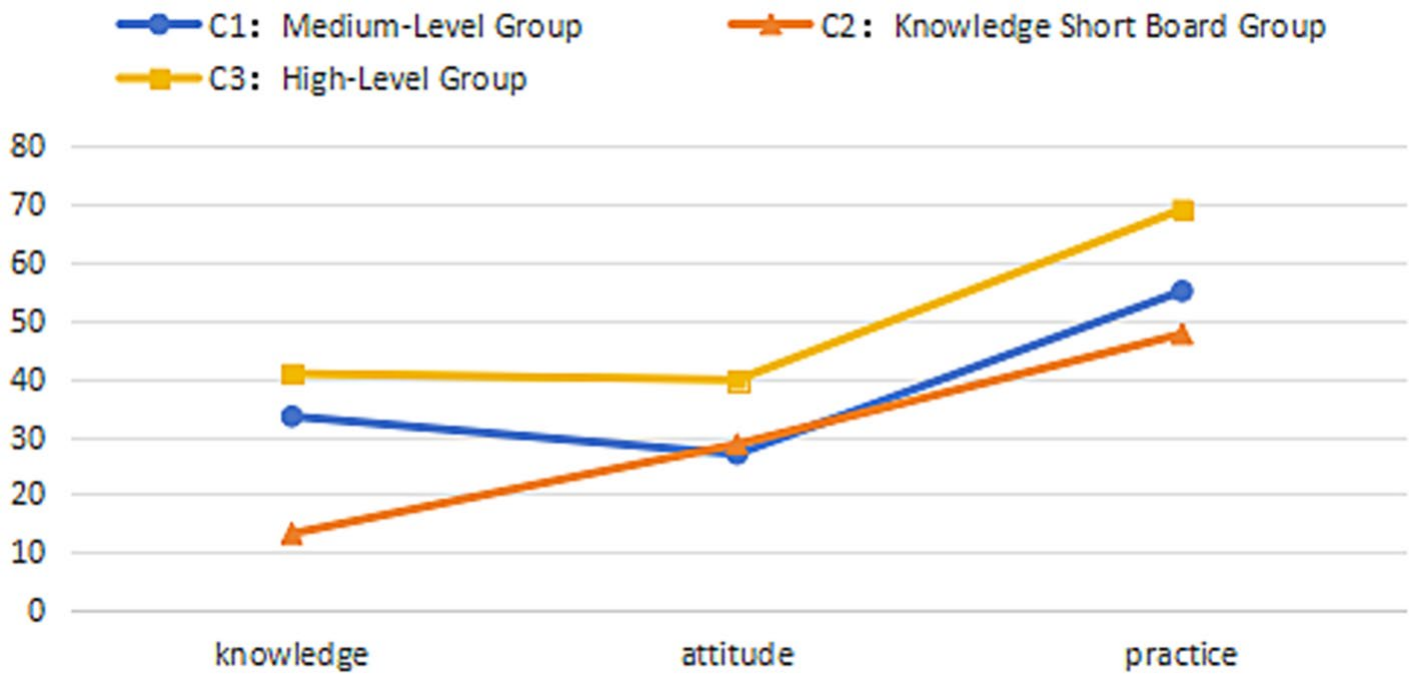
Potential profile analysis of medical device alarm fatigue KAP status.

Latent profile analysis was conducted based on the mean scores of the three dimensions of KAP. Starting from a single category, the number of categories was increased successively, and four latent profile models were fitted. The results are shown in Table 2. In models 2, 3, and 4, the values of AIC, BIC, and a BIC decreased progressively, and both LMR and BLRT reached significant levels (*p* < 0.05). The 4-profile model, though statistically viable, included a negligible subgroup (2.7%) with no distinct clinical or empirical rationale. This group’s scores did not deviate meaningfully from adjacent profiles (e.g., overlapping confidence intervals in practice dimension scores), suggesting it lacked substantive uniqueness. In contrast, the 3-profile solution cleanly differentiated nurses into high-, medium-, and knowledge deficit groups, each with practical implications for training and policy (Figure 2). The negligible size and indistinct characteristics of the fourth group further supported its exclusion. According to the external characteristics of each dimension of the scale, the categories were named as follows. In category C1, the KAP scores were roughly between C2 and C3, so it was named the “Medium-Level” group. In category C2, the knowledge dimension was at the lowest level compared with the other two categories, so it was named the “Knowledge shortfall” group. In category C3, all dimensions were at a higher level, so it was named the “High-Level” group.

**Table 2:** Summary of potential profile model fitting information for medical device alarm fatigue KAP.

Model	AIC	BIC	aBIC	Entropy	*P*-value		Category probability (%)
					LMR	BLRT	
1	23885.49	23915.97	23896.92	–	–	–	–
2	21738.62	21789.41	21757.65	1.000	<0.001	<0.001	84.1/15.9
3	20587.89	20659.00	20614.53	0.995	<0.001	<0.001	73.9/10.3/15.8
4	20460.09	20552.52	20492.35	0.988	<0.001	<0.001	2.7/7.8/15.9/73.6

The 3-profile model was selected based on high entropy, meaningful subgroup sizes (all >10%), and theoretical interpretability. AIC, Akaike Information Criterion; BIC, Bayesian Information Criterion; aBIC, sample-size-adjusted BIC; LMR, Lo–Mendell–Rubin test; BLRT, Bootstrapped Likelihood Ratio Test.

### 3.4 Univariate analysis of KAP level of medical device alarm fatigue

There were no statistically significant differences among the three latent profile categories of KAP level of medical device alarm fatigue in terms of marital status, number of children, hospital attributes, and monthly income (*p* > 0.05). Statistically significant differences were found in other factors (*p* < 0.05) (see Table 1 for details).

### 3.5 Multivariate analysis of latent profiles of KAP level

The variables with statistical significance in the univariate analysis were included as independent variables in a multinomial logistic regression model, with the three latent profile categories as the dependent variable (using the high-level group as the reference category). The results of the multinomial logistic regression analysis are presented in Table 3. Several factors were significantly associated with profile membership relative to the High-Level group. Compared with the high-level group, nurses with 1–5 years of experience had significantly higher odds of being in the medium-level group (OR = 2.64, 95% CI: 1.05–6.65). Participation in alarm-related knowledge training was associated with significantly lower odds of being in the medium-level group (OR = 0.35, 95% CI: 0.19–0.65) and the knowledge-deficit group (OR = 0.25, 95% CI: 0.10–0.66) compared with the high-level group. A one-point increase in psychological resilience score was associated with approximately 21% lower odds of being in the medium-level group (OR = 0.79, 95% CI: 0.76–0.82) and 20% lower odds of being in the knowledge-deficit group (OR = 0.79, 95% CI: 0.76–0.83), relative to the high-level group.

**Table 3:** Regression analysis of potential profile categories for medical device alarm fatigue KAP.

Item	Medium level group				Knowledge-deficit group			
	*β*	*P*	OR	95%CI	*β*	*P*	OR	95%CI
Age								
<30	2.054	0.004	7.796	1.946–31.231	2.834	0.065	17.014	0.838–345.559
31–40	1.057	0.105	2.879	0.802–10.331	−0.055	0.972	0.947	0.046–19.503
41–50	−0.186	0.769	0.831	0.240–2.872	−1.175	0.472	0.309	0.013–7.608
Hospital level								
Tertiary	−0.032	0.981	0.969	0.069–13.640	−3.024	0.041	0.049	0.003–0.879
Secondary	−0.723	0.601	0.485	0.032–7.312	−4.188	0.006	0.015	0.001–0.297
Years of experience in hemodialysis work								
1–5	0.971	0.039	2.641	1.050–6.647	−0.200	0.854	0.818	0.097–6.915
6–10	0.487	0.273	1.627	0.682–3.883	0.638	0.554	1.893	0.229–15.667
11–15	0.349	0.440	1.418	0.585–3.436	−0.330	0.793	0.719	0.061–8.430
Have participated in medical equipment alarm related knowledge training	−1.052	0.001	0.349	0.188–0.648	−1.370	0.005	0.254	0.097–0.663
Have participated in the analysis of medical device alarm adverse events	−0.078	0.749	0.925	0.572–1.494	−1.256	0.004	0.285	0.122–0.667
Total score of psychological resilience	−0.238	< 0.001	0.788	0.759–0.817	−0.231	< 0.001	0.794	0.759–0.830

Variables that were significant in univariate analysis (Table 1) but excluded from the final regression model due to multicollinearity or loss of significance after adjustment include: gender, educational level, professional title, CRRT shift, and hemodialysis specialist nurse status. See Supplementary Table S1 for the full model with all variables and variance inflation factor (VIF) values.9.

## 4 Discussion

This study employed LPA to identify three distinct KAP profiles among hemodialysis nurses regarding medical device alarm fatigue. The overall KAP level was moderate, which is consistent with findings from ICU nurses in China (15, 17), suggesting possible gaps in alarm management training across specialties. The lower knowledge scores relative to practice may reflect limited formal education on alarm systems in Chinese hospitals, where alarm fatigue research and management started later than in Western settings (18). This may be related to the late start of research and management of alarm fatigue in China. The Emergency Care Research Institute (ECRI) in the United States has defined alarm fatigue as the top health technology hazard for three consecutive years (19). The lack of relevant training in hospitals leads to insufficient knowledge reserves of nurses regarding alarms (20). In clinical practice, nurses are continuously exposed to equipment alarms. Frequent false alarms can erode their trust in alarm systems, thereby increasing the risk of alarm fatigue (21). These findings suggest that there may be room for improvement in nurses’ knowledge and attitudes toward alarm management, and highlight the potential value of developing targeted educational strategies, though further intervention studies are needed to establish their effectiveness.

The three profiles—medium-level (73.8%), knowledge-deficit (10.3%), and high-level (15.9%)—underscore significant heterogeneity. Younger, less experienced nurses were more likely to belong to the medium-level group, possibly due to fewer training opportunities. Compared with the high-level group, nurses working in higher-grade hospitals had lower odds of being in the knowledge-deficit group. Similarly, participation in alarm-related training or adverse event analysis, and higher resilience scores were associated with lower odds of being in either the medium-level or knowledge-deficit groups, relative to the high-level group. These factors highlight the potential influence of institutional resources, structured education, and psychological resilience (22).

Training and adverse event analysis emerged as modifiable factors linked to better KAP outcomes. Systematic training may support alarm knowledge acquisition and could contribute to more attentive attitudes (23). Learning from real adverse events is associated with enhanced risk awareness and safety culture (24). Therefore, integrating case-based learning and simulation into continuing education could be beneficial.

Resilience was strongly associated with KAP profile membership. Hemodialysis nurses work in a high-stress environment, independently operating complex equipment and managing emergent complications (22). Research has shown that using adverse event analysis as a learning tool may enhance risk awareness and safety consciousness (23). Therefore, in clinical practice, It may be beneficial to explore targeted training strategies for less experienced nurses, with the goal of improving their alarm-related knowledge and practices—an approach that should be validated through prospective intervention studies. This can be achieved through mentorship programs, team-based learning, and quality improvement circle activities (24, 25), all of which can enhance the KAP level of medical device alarm fatigue among nurses.

The study revealed that higher psychological resilience was associated with lower odds of being in the medium-level or knowledge-deficit groups, suggesting that resilient nurses were more likely to belong to the high-level KAP group. Psychological resilience refers to an individual’s ability to adapt well to various pressures and setbacks (16). Hemodialysis nurses, as a special group of caregivers, are required to operate dialysis machines independently and manage various emergencies and complications related to dialysis. The complex procedures and the closed nature of the working environment place a heavy psychological burden on these nurses (26). Research has shown that psychological burden positively predicts alarm fatigue among nurses (27). Psychological resilience acts as a protective resource for individuals, promoting positive adaptation and maintaining mental health in high-risk environments (28). While our cross-sectional design precludes causal claims, longitudinal studies could test whether resilience-building interventions e.g., mindfulness programsimprove KAP (29). Although causal direction cannot be inferred, resilience-building interventions (e.g., mindfulness, peer support) could be integrated into alarm management programs to support nurse wellbeing.

Several limitations should be acknowledged. First, all primary measures were self-reported, subjecting the study to social desirability bias, recall bias, and common method variance (30–33). Although we implemented anonymity and attention checks, future studies should incorporate objective measures (e.g., alarm log data, direct observation). Second, convenience sampling within a single province limits generalizability; the overrepresentation of tertiary hospitals (70.6%) may not reflect primary settings. Third, the cross-sectional design precludes causal inference. Fourth, unmeasured confounders (e.g., unit-level alarm policies, staffing levels, alarm system characteristics) may influence profile membership. Fifth, while the 3-profile solution demonstrated high entropy (0.995), LPA remains probabilistic with residual classification uncertainty.

## 5 Conclusion

This study moves beyond variable-centered approaches by applying latent profile analysis to reveal significant heterogeneity in KAP levels regarding medical device alarm fatigue among hemodialysis nurses. Three distinct profiles were identified: medium-level, knowledge-deficit, and high-level. Compared with the high-level group, younger age, less experience, lack of specific training, lower hospital grade, and lower resilience were associated with higher odds of belonging to the medium-level or knowledge-deficit groups.

These findings support a precision education model in which interventions are tailored to specific nurse profiles: For the knowledge-deficit group, focus on foundational knowledge through mandatory e-learning and simulation. For the medium-level group, reinforce knowledge and attitudes via in-service training and practical tools (e.g., alarm checklists). For the high-level group, leverage their expertise by involving them as safety champions and mentors.

Integrating resilience-building components into alarm management programs may also support nurses’ adaptive capacity. This person-centered approach offers a potential pathway for mitigating alarm fatigue, supporting nurse wellbeing, and enhancing patient safety in hemodialysis units. Future intervention studies are needed to evaluate the efficacy of these tailored strategies.

## Data Availability

The raw data supporting the conclusions of this article will be made available by the authors, without undue reservation.
